# WIDOWHOOD AND COGNITIVE DECLINE: THE ROLES OF FRIENDS AND GENDER

**DOI:** 10.1093/geroni/igad104.1587

**Published:** 2023-12-21

**Authors:** Zhenmei Zhang, Hui (Cathy) Liu, Ning Hsieh

**Affiliations:** Michigan State University, East Lansing, Michigan, United States; Purdue University, West Lafayette, Indiana, United States; Michigan State University, East Lansing, Michigan, United States

## Abstract

Widowhood has been identified as a risk factor for cognitive decline and dementia. Yet less is known about the protective and exacerbating factors of cognitive decline following widowhood. This study examines the roles of friendship characteristics in moderating the negative effect of spousal loss on cognition for older Americans. Drawing on data from the 2006-2018 Health and Retirement Study, we aim to answer two questions: 1) whether friendship network and quality moderate the association between spousal loss and cognitive decline in late life; 2) whether the moderating effect of friendship characteristics on cognition differ for men and women. Cognitive trajectories were assessed every other year from 2006 to 2018 (in total 7 waves). Friend characteristics were assessed in the 2006 psychosocial questionnaire and included the number of close friends, contact frequency, and support and strain from friends. The analytical sample comprised 3,636 respondents aged 50 and older who were married in 2006 and participated in the psychosocial questionnaire. Among the final sample, 370 lost spouses between 2006 and 2010, and 3,266 remained married throughout the observation period. Preliminary results from latent growth curve models showed that several characteristics of friendship including contact frequency and strain from friends are significantly associated with cognitive function at baseline. Furthermore, strain from friends interacted with widowhood status and gender to shape cognitive trajectories. Specifically, higher friendship strain was associated with a faster cognitive decline for the widowed but not for the married a pattern observed only among men but not women

